# Dynamics and Predictors of Cognitive Impairment along the Disease Course in Multiple Sclerosis

**DOI:** 10.3390/jpm11111107

**Published:** 2021-10-28

**Authors:** Elisabet Lopez-Soley, Eloy Martinez-Heras, Magi Andorra, Aleix Solanes, Joaquim Radua, Carmen Montejo, Salut Alba-Arbalat, Nuria Sola-Valls, Irene Pulido-Valdeolivas, Maria Sepulveda, Lucia Romero-Pinel, Elvira Munteis, Jose E. Martínez-Rodríguez, Yolanda Blanco, Elena H. Martinez-Lapiscina, Pablo Villoslada, Albert Saiz, Elisabeth Solana, Sara Llufriu

**Affiliations:** 1 Center of Neuroimmunology, Laboratory of Advanced Imaging in Neuroimmunological Diseases, Hospital Clinic Barcelona, Institut d’Investigacions Biomediques August Pi i Sunyer (IDIBAPS) and Universitat de Barcelona, 08036 Barcelona, Spain; elopez2@clinic.cat (E.L.-S.); emartind@clinic.cat (E.M.-H.); magiandorra@gmail.com (M.A.); c.montejo.gonzalez@gmail.com (C.M.); salba@clinic.cat (S.A.-A.); nuria.sola@grupsagessa.com (N.S.-V.); irenepulidovaldeolivas@gmail.com (I.P.-V.); msepulve@clinic.cat (M.S.); yblanco@clinic.cat (Y.B.); elenahmlapiscina@gmail.com (E.H.M.-L.); pvillos@stanford.edu (P.V.); asaiz@clinic.cat (A.S.); 2 Imaging of Mood- and Anxiety-Related Disorders (IMARD) Group, IDIBAPS and CIBERSAM, 08036 Barcelona, Spain; solanes@clinic.cat (A.S.); radua@clinic.cat (J.R.); 3 Centre for Psychiatry Research, Department of Clinical Neuroscience, Karolinska Institutet, Solna, 171 77 Stockholm, Sweden; 4 Early Psychosis: Interventions and Clinical-Detection (EPIC) Laboratory, Department of Psychosis Studies, Institute of Psychiatry, Psychology and Neuroscience, King’s College London, London WC2R 2LS, UK; 5 Multiple Sclerosis Unit, Neurology Department, Hospital Universitari de Bellvitge, IDIBELL, 08907 Barcelona, Spain; lromeropinel@gmail.com; 6 Neurology Department: Hospital del Mar Medical Research Institute (IMIM), 08003 Barcelona, Spain; emunteis@parcdesalutmar.cat (E.M.); jemartinezrguez@gmail.com (J.E.M.-R.)

**Keywords:** cognition, cognitive impairment, neuroimaging, longitudinal, predictors, multiple sclerosis

## Abstract

(1) Background: The evolution and predictors of cognitive impairment (CI) in multiple sclerosis (MS) are poorly understood. We aimed to define the temporal dynamics of cognition throughout the disease course and identify clinical and neuroimaging measures that predict CI. (2) Methods: This paper features a longitudinal study with 212 patients who underwent several cognitive examinations at different time points. Dynamics of cognition were assessed using mixed-effects linear spline models. Machine learning techniques were used to identify which baseline demographic, clinical, and neuroimaging measures best predicted CI. (3) Results: In the first 5 years of MS, we detected an increase in the z-scores of global cognition, verbal memory, and information processing speed, which was followed by a decline in global cognition and memory (*p* < 0.05) between years 5 and 15. From 15 to 30 years of disease onset, cognitive decline continued, affecting global cognition and verbal memory. The baseline measures that best predicted CI were education, disease severity, lesion burden, and hippocampus and anterior cingulate cortex volume. (4) Conclusions: In MS, cognition deteriorates 5 years after disease onset, declining steadily over the next 25 years and more markedly affecting verbal memory. Education, disease severity, lesion burden, and volume of limbic structures predict future CI and may be helpful when identifying at-risk patients.

## 1. Introduction

Multiple sclerosis (MS) is a chronic inflammatory demyelinating disease of the central nervous system that entails physical and cognitive impairment (CI). The latter has been reported in 40–70% of people with MS and it has a severe impact on the individual’s quality of life [1,2]. CI can be detected in the early phases of MS, but it is more frequent as overall disability accrues [3]. The pattern of cognitive decline predominantly affects information processing speed (IPS) and episodic memory, although executive functions, semantic fluency, and visuospatial analysis may also be altered [4,5]. However, how this deterioration evolves and affects different cognitive domains as the disease progresses is still to be determined. 

A few longitudinal studies have investigated the association between clinical and imaging features of MS with cognitive decline, suggesting a predictive value of baseline cognitive status [5], baseline IPS [6], education, and aging [7]. Using different magnetic resonance imaging (MRI) techniques, a relationship has been demonstrated between CI and the combined effect of white matter (WM) and gray matter (GM) damage [8]. In addition, identifying neurodegeneration in specific and cognitively relevant GM regions may help to more accurately predict CI.

Characterizing the natural course of cognitive performance in MS, and identifying predictors of CI, are still distant milestones in clinical MS research. Therefore, in this study, we first describe the temporal dynamics of global cognition and cognitive domains using mixed-effects models, which allowed us to obtain model estimates of specific parameters and to control for between- and within-subject variability. Subsequently, we investigated the baseline demographic, clinical, and MRI measures that best predicted the CI using machine learning (ML) techniques. These issues were addressed in an appropriately large cohort of MS patients with a wide range of disease duration.

## 2. Materials and Methods

### 2.1. Participants, Clinical, and Cognitive Assessment

For this longitudinal study, we collected data from a prospective cohort recruited at the MS Unit of the Hospital Clinic of Barcelona from January 2011 to February 2020 [9,10]. The criteria for inclusion in this study were aged between 18 and 65 years, and having at least two clinical and cognitive assessments, with MRI scans available at the first evaluation. Patients did not present any relapse or received any corticosteroid treatment in the last 30 days of the study visit. As such, 212 MS patients fulfilled the inclusion criteria and were analyzed. We collected data regarding sex, age, educational level, disease duration, disease type, the number of relapses before study inclusion, the use of disease-modifying therapies (DMTs), and their global disability evaluated with the Expanded Disability Status Scale (EDSS) [11]. The Ethics Committee at the Hospital Clinic of Barcelona approved the study, and all the participants provided their signed informed consent prior to their enrolment.

At each visit, the participants underwent a neuropsychological evaluation using the Rao’s battery [12], with alternate versions when available. Raw values were transformed into z-scores by adjusting for age and education according to the Spanish normative data, and they were grouped in terms of global cognition and for each cognitive domain (verbal and visual memory, attention-IPS, and semantic fluency) [13]. Failure in any test was considered when z-score was below −1.5 standard deviation (SD) of the norm. CI in a given cognitive domain was defined as a failure in at least one test assessing that domain, and global CI was defined as an impairment in at least two cognitive tests evaluating the same or different cognitive domains.

### 2.2. Magnetic Resonance Imaging (MRI)

#### 2.2.1. MRI Acquisition and Processing

Baseline MRI were acquired on a 3 Tesla Magnetom Trio (SIEMENS, Erlanger, Germany) scanner using a 32-channel phased-array head coil, as described previously [10]. Two different acquisition protocols were used, involving a 3D-Magnetization Prepared Rapid Acquisition Gradient Echo (MPRAGE) and 3D-T2 fluid attenuated inversion recovery (FLAIR) sequence (see Supplementary Material).

#### 2.2.2. Structural MRI Processing for Volumetric Analysis

WM lesions were defined semi-automatically into the 3D-MPRAGE space with the registered 3D-FLAIR image as a reference to improve lesion identification using the Jim7 Software (http://www.xinapse.com/j-im-7-software/). Lesion in-painting was applied to the 3D-MPRAGE image to enhance segmentation and registration. GM regions were parcellated using the Mindboggle software (https://mindboggle.info), applying the Desikan–Killiany Tourville cortical labeling atlas, and the automated subcortical segmentation was achieved with the FSL-FIRST package (fsl.fmrib.ox.ac.uk/fsl/fslwiki/FIRST), resulting in 31 cortical and 7 subcortical labels per hemisphere [14,15]. The volumetric measurements were analyzed using the SIENAX [16] scaling factor to reduce the head-size variability. 

We removed interscan variability between the different acquisition protocols using the ComBat function in the R software [17,18].

### 2.3. Statistical Analysis

All baseline demographic, clinical, and neuroimaging data were described through the median and interquartile range (IQR) or the mean (±SD) for quantitative variables as appropriate as well as through the absolute numbers and the proportions of the qualitative variables. The normal distribution of the data was checked by histograms inspection and using the Shapiro–Wilks test.

We used mixed-effects linear regression to model the dynamics of cognition throughout the course of MS. Models adjusted for age at MS onset, educational level, and sex were used to fit the rates of global cognitive performance and of each cognitive domain using disease duration as a main fixed-effect predictor. In addition, we used linear spline models with the same variables as in the mixed-effects regressions to divide the duration of the disease into three periods. Using visual inspection of the raw data together with prior evidence [19] and the Akaike Information Criterion [20], we selected knots at 5 and 15 years of disease duration to model our data. These models provided three parameters, beta coefficients, for the change in cognition relative to disease duration.

We used ML techniques to identify which baseline demographic, clinical, and MRI measures best serve as predictors of CI. A priori, potential predictor variables were sex, educational level (basic, primary, secondary, or higher), disease duration, disease type, EDSS score, use of DMTs (yes or no), number of relapses before study inclusion, lesion volume, and 76 cortical and subcortical regional volumes [15]. Multiple imputation was employed to handle missing data: we used multiple regression to find the variable distribution and we replaced the missing value by taking a random value from the distribution found. Logistic Lasso regressions were performed to predict the global cognitive status (preserved or impaired, see above). The effect of age was removed from the anatomical brain features, although we included age as a predictor variable in the Lasso model. Lasso regressions automatically select a small number of baseline measures, avoiding overfitting. To validate the performance of the ML models, we used a 10-fold cross-validation method, splitting the overall sample into training and test datasets. We created the imputation algorithms and Lasso regressions using the training datasets alone, while we assessed the performance of the predictions in the independent test datasets. Due to the use of multiple imputation and folds, we created several ML models. We selected the most representative model as the one with the highest overlaps (Dice coefficient) with the other models in the selection of the baseline measures. The same procedure was used for each specific cognitive domain.

All the analyses were performed using the R statistical software (version 3.6.0, www.R-project.org), and the statistical significance was set at *p* < 0.05. 

## 3. Results

A cohort of 212 MS patients who performed a median of three clinical visits per patient (range, 2–5; total assessments = 605) with a median follow-up time of 2.1 years (range 0.9–7.9 years) were included in this study. In terms of the baseline characteristics (Table 1), the patients were mostly female (68%), middle aged-adults (41 ± 9.47 years), with a relapsing-remitting MS (83%) and with a median disease duration of 8.2 years (range, 0.1–29.0).

One hundred and eleven patients (52%) were receiving DMTs at baseline, and from them, 94 patients (85%) used moderate-efficacy DMTs (Table S1).

At the latest follow-up, 77 patients (36%) had global CI, 58 patients (27%) had verbal memory impairment, 51 patients (24%) had visual memory impairment, 38 patients (18%) had attention-IPS impairment, and 41 patients (20%) had semantic fluency impairment. 

### 3.1. Cognitive Trajectory throughout Disease Course

**Figure 1:**
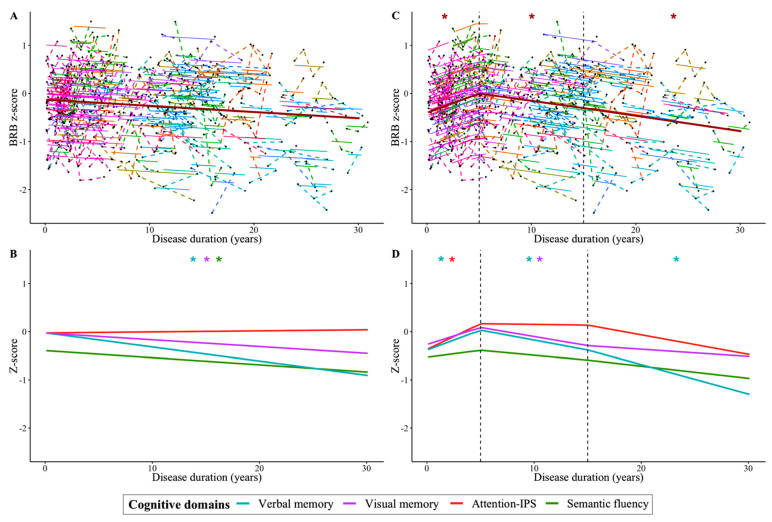
Dynamics of cognitive performance in MS as the disease progresses. The global cognition z-score (**A**) and cognitive domains z-score (**B**) were modeled by mixed-effect regressions. The duration of the disease was divided into three periods by spline models with two knots (at 5 and 15 years of disease duration) represented by dotted black vertical lines (for the global cognition z-score (**C**) and each domain z-score (**D**). Black points joined by a broken line represent the individual trajectories of the changes in the global cognition z-scores, the continuous lines represent the individual fit of the model, and the thicker brown line represents the population model (**A**,**C**). Population model lines of cognitive domains are differentiated by color (**B**,**D**): blue for verbal memory, purple for visual memory, red for attention-IPS, and green for semantic fluency. The x-axis represents the time in years from clinical onset. All models were fitted using the lme4 package in R version 3.5.2 (R Foundation for Statistical Computing: * *p* < 0.05).

According to the linear mixed-effects models, there was an annual cognitive decline that affected verbal memory, visual memory, and semantic fluency (Figure 1A, Figure 1B, and Table S2). A trend was found in global cognition (*p* = 0.058), and no significant model was found for attention-IPS (*p* = 0.345). 

When we divided the duration of the disease into three periods, we detected distinct cognitive slopes for each stage (Figure 1C,D and in Table S3). The initial period extended over the first 5 years of the disease, during which an increase in cognition was evident. In the second period, covering 5–15 years of the disease and the third phase, 15–30 years, the cognitive decline in the participants became increasingly accentuated. In the first 5 years of MS, we detected an enhancement in global cognition (β = 0.080 (95% CI, 0.04 to 0.12) z-score/year; *p* = <0.001), verbal memory (β = 0.083 (95% CI, 0.01 to 0.16) z-score/year; *p* = 0.037), and attention-IPS (β = 0.107 (95% CI, 0.05 to 0.16) z-score/year; *p* = <0.001). However, this trajectory was followed by a decline in global cognition (β = −0.029 (95% CI, −0.05 to −0.01) z-score/year; *p* = 0.013), verbal memory (β = −0.041 (95% CI, −0.08 to 0.00) z-score/year; *p* = 0.047), and visual memory (β = −0.041 (95% CI, −0.08 to −0.01) z-score/year; *p* = 0.024) between 5 and 15 years of the disease. Moreover, similar dynamics were observed during the 15–30 years of MS course, during which cognitive decline continued in global cognition (β = −0.031 (95% CI, −0.06 to −0.01) z-score/year; *p* = 0.021) and verbal memory (β = −0.055 (95% CI, −0.10 to −0.01) z-score/year; *p* = 0.018), and a trend was observed toward a decline in attention-IPS (β = −0.035 (95% CI, −0.07 to 0.00) z-score/year; *p* = 0.055). No significant effect was detected on semantic fluency performance. 

### 3.2. Demographic, Clinical, and MRI Baseline Predictors of Future CI 

A Lasso regression was employed to predict CI at the latest follow-up. The models that showed the strongest performance were verbal memory (positive predictive value (PPV) = 62%; negative predictive value (NPV) = 90%) and attention-IPS (PPV = 38%; NPV = 92%), which were more accurate (79% and 73%, respectively) in predicting CI than the other models (Table 2).

The resulting predictive model of global CI included educational level, disease duration, EDSS score, and the number of previous relapses as clinical parameters. The model also included lesion volume and six cortical regional volumes, covering the bilateral parahippocampus, left hippocampus, and right caudate entorhinal and rostral anterior cingulate (Table 3).

In terms of verbal memory, CI was predicted by educational level, disease type, EDSS score, and the number of previous relapses. The MRI predictors identified involved lesion volume and six cortical regional volumes, including the right parahippocampus and rostral anterior cingulate, and the pars opercularis, pericalcarine, thalamus, and accumbens of the left hemisphere. Lesion volume was the only predictor selected for CI in terms of visual memory. CI in attention-IPS was predicted by the EDSS, lesion volume and volume of the right hippocampus, caudal anterior cingulate and entorhinal, and the left pericalcarine. The prediction of semantic fluency CI was explained by a model that involved lesion volume and the volume of the left hippocampus and the right rostral anterior cingulate.

## 4. Discussion

In this longitudinal study, we set out to better understand the deterioration of cognition in MS by describing its temporal dynamics and by identifying predictors of CI. The results reveal different patterns of worsening over the disease course, both in terms of global cognition and the distinct cognitive domains, suggesting a progressive decline after the first 5 years of disease onset that most markedly affects verbal memory. When focusing on the five models that best predicted CI, we found that verbal memory and attention-IPS models had the strongest predictive performance. The results reinforce the importance of the educational level, disease severity, lesion load, and certain GM regional volumes, mainly involving the medial temporal lobe areas and the cingulate, as predictors of cognitive deficits.

There have been some attempts to describe the evolution of cognitive performance in patients with MS, mainly focusing on short time follow-up periods [3,5]. However, the diversity of cohort characteristics and the use of disparate range of cognitive tests and criteria for diagnosing impairment has produced quite heterogeneous data that prove to be difficult to compare across studies. Here, we characterized temporal modifications to different cognitive domains in a cohort of patients with wide ranging disease duration. Our data showed a progressive decline as opposed to an abrupt development of CI, supporting a combined role of age, neurodegeneration, the exhaustion of cognitive reserve, and a loss of plasticity in this clinical manifestation of MS [21]. Moreover, we modeled the trajectory in three different periods by providing differential slopes of the cognitive change during the course of the disease. The results showed an increase in global cognition, verbal memory, and attention-IPS in the first five years after MS onset, which was followed by a decline in cognitive performance. This is a surprising finding even though it is consistent with previous data indicating that cognitive deterioration occurs mainly after the fifth year following disease onset [22]. Several explanations may account for the former. First, it may reflect the capacity of the brain to compensate for the pathological effects of MS lesions through its cognitive reserve, which is a response that may be particularly protective in early stages before structural damage accumulates. Second, the mood disorders such as anxiety or depressive symptoms associated with the diagnosis of MS may negatively affect the results of a first cognitive assessment [23]. Finally, there might be a possible effect of learning in the retesting of cognition that could be present at any stage of the disease, even though we used alternate forms of the tests at each evaluation whenever this was possible.

The cognitive trajectory from the fifth year after MS onset onwards was driven by a decline in verbal and visual memory, although only verbal memory continued to deteriorate until the 30th year of the disease, along with a trend for attention-IPS to decline. Our data reinforce other smaller longitudinal studies, where CI was driven by evolving dysfunction in verbal memory and IPS [5]. By contrast, it was recently shown that IPS was the first domain to be affected [24]. This incongruence may reflect methodological differences, as we grouped the results from the attention-IPS tests into a single cognitive domain, and our cohort also had a lower educational level. Moreover, we cannot rule out a contribution of the distinct cognitive phenotypes in MS [25], as they may differ between cohorts of patients.

Little is known about what may serve to predict the development of CI, hampering research into early prevention and treatment. In the present analysis, the verbal memory and the attention-IPS prediction models produced the highest predictive balanced accuracy and a very high NPV. Educational level was a predictor in the global cognition and verbal memory models, which might reflect the protective role of cognitive reserve in CI [26,27]. In addition, the disease severity indicated by the EDSS and the number of previous relapses seemed to be related to future impairment in global cognition, verbal memory, and attention-IPS models.

Regarding MRI features, global lesion volume was selected as a predictor in all models. In fact, lesion accumulation has been associated with more severe cognitive dysfunction [28] by promoting brain network disruption [10]. Even so, the present results enabled the identification of the specific cortical regional areas related to future cognition. The hippocampus influences global cognition, verbal memory, and semantic fluency, which is consistent with the theory that it is an integral component of the brain network that supports verbal memory and word generation [25,29,30,31]. Similarly, the volume of the anterior cingulate cortex was present in all predictive models, except for the visual memory model. This region is involved in the fronto-parietal network, and it plays a key role in executive functions, as well as participating in the working memory network [32,33]. Moreover, the thalamus, a highly connected nucleus, has been associated with learning and memory function, and it seems to be a good predictor for CI in MS [5,34], although here, it was more specifically associated with verbal memory impairment. All these areas are part of the limbic system, which plays a crucial role in various cognitive functions [35].

This study has several strengths, including the fact that participants were prospectively and consecutively recruited, thereby preventing a selection bias and enhancing the generalizability of the results. Drawing up a global pattern of cognition in MS was only possible because our cohort included patients with a clinical disease duration of up to 30 years. In addition, all the analyses were performed for global and stratified cognition. Our study also has some limitations. Working with a real-world MS cohort implies that it is predominantly composed of relapsing-remitting MS patients, the most common phenotype encountered clinically in the current treatment era. Moreover, we were unable to assess the influence of mood disorders and fatigue on cognition because, unfortunately, the protocol did not include any mood or fatigue specific test. Furthermore, we do not have a matched control group, although we used z-scores based on normative data to address the changes in cognition that can be expected in accordance with age and educational level. In addition, it has not been possible to analyze the effect of DMTs on cognition, as the predictive models could be influenced by the low proportion of treated patients (52%) at the study initiation predominantly using moderate-efficacy DMTs. Finally, the inclusion of GM lesion volume, WM lesion location, or advanced quantitative MRI measures, such as functional and diffusion MRI, in future studies might be useful to improve our understanding of cognition and its MRI related factors in MS.

## 5. Conclusions

In conclusion, cognition in MS patients progressively deteriorates after the first 5 years of the disease, with a steady decline over the next 25 years that affects verbal memory more markedly. Moreover, CI is predicted by the educational level, disease severity, lesion load, and volume of high-order regions, including the hippocampus and anterior cingulate cortex, with a strong NPV for the verbal memory and attention-IPS in particular. Consequently, beneficial cognitive maintenance strategies should be adopted that focus on predictors that identify patients at risk of CI and which promote activities such as intellectual enrichment that attenuate the impact of brain burden in the initial years of the disease as an adequate treatment window.

## Figures and Tables

**Table 1:** Demographic, clinical, and MRI characteristics of MS patients at baseline.

	Entire Cohort (*n* = 212)
Female, *n* (%)	145 (68)
Age, mean (SD)	41 (9.47)
Educational level, *n* (%)	
Basic (0–8 years)	16 (8)
Primary (9–12 years)	85 (40)
Secondary (13–16 years)	75 (35)
Higher (>17 years)	36 (17)
Disease duration, median (range)	8.20 (0.1–29.0)
Disease type, *n* (%)	
Clinically isolated syndrome	19 (9)
Relapsing-remitting MS	176 (83)
Secondary progressive MS	13 (6)
Primary progressive MS	4 (2)
EDSS score, median (range)	2.0 (0–7.0)
Use of DMTs, *n* (%)	111 (52)
Number of previous relapses, median [IQR]	3 (2–4)
Lesion volume (cm^3^), median [IQR]	5.16 (2.37–12.15)

The data represent the absolute numbers and the proportions of the qualitative data, or the median and the interquartile range (IQR) for the quantitative data, unless otherwise specified. SD: standard deviation; MS: multiple sclerosis; EDSS: Expanded Disability Status Scale; DMTs: Disease-Modifying Therapies.

**Table 2:** Performance evaluation of each Lasso regression model.

Cognitive Domain	*N*	Balanced Accuracy (%)	Sensitivity (%, 95% CI)	Specificity (%, 95% CI)	PPV (%, 95% CI)	NPV (%, 95% CI)
Global cognition	212	71	70 (59–80)	71 (63–79)	58 (47–68)	81 (72–87)
Verbal memory	212	79	76 (63–86)	82 (76–88)	62 (50–73)	90 (84–94)
Visual memory	212	62	71 (56–82)	54 (46–62)	33 (24–42)	85 (77–91)
Attention-IPS	212	73	71 (54–85)	75 (68–81)	38 (27–50)	92 (86–96)
Semantic fluency	210	62	51 (44–59)	73 (57–86)	89 (81–94)	29 (19–36)

Balanced Accuracy is defined as the arithmetic mean of sensitivity and specificity. Sensitivity is defined as the proportion of subjects who developed cognitive impairment that are correctly classified. Specificity is defined as the proportion of subjects who did not develop cognitive impairment that are correctly classified. The predictive model of cognitive impairment in semantic fluency was generated with 210 patients. CI: confidence interval; PPV: positive predictive value; NPV: negative predictive value; IPS: information processing speed.

**Table 3:** Predictors of each Lasso regression model.

Cognitive Domain	*N*	Predictors	β	Predictors Selection Rates (Frequency *, %)
Global cognition	212	Educational level	−0.060	1253 (63)
		Disease duration	0.034	936 (47)
		EDSS score	0.325	2000 (100)
		Number of previous relapses	0.069	1635 (82)
		Lesion volume	0.388	2000 (100)
		LH parahippocampal	0.127	1793 (90)
		Left hippocampus	0.070	1595 (80)
		Right caudate	−0.057	1133 (57)
		RH entorhinal	0.044	1087 (54)
		RH parahippocampal	0.085	1836 (92)
		RH rostral anterior cingulate	0.195	1984 (99)
Verbal memory	212	Educational level	−0.386	1983 (99)
		Disease type	0.229	1557 (78)
		EDSS score	0.458	2000 (100)
		Number of previous relapses	0.115	1935 (97)
		Lesion volume	0.309	1998 (100)
		LH parsopercularis	−0.101	1046 (52)
		LH pericalcarine	0.226	1894 (95)
		Left thalamus proper	−0.096	1536 (77)
		Left accumbens area	0.038	1102 (55)
		RH parahippocampal	0.680	2000 (100)
		RH rostral anterior cingulate	0.040	1407 (70)
Visual memory	212	Lesion volume	0.054	1949 (97)
Attention-IPS	212	EDSS score	0.654	2000 (100)
		Lesion volume	0.199	1975 (99)
		LH pericalcarine	0.103	1838 (92)
		Right hippocampus	0.078	0.919 (83)
		RH caudal anterior cingulate	0.035	881 (44)
		RH entorhinal	0.111	1275 (64)
Semantic fluency	210	Lesion volume	−0.019	1005 (50)
		Left hippocampus	−0.017	1071 (53)
		RH rostral anterior cingulate	−0.021	658 (33)

The demographic, clinical, and MRI variables that remained in the age-adjusted predictive model of cognitive impairment in each domain are shown. EDSS: Expanded Disability Status Scale; RH: right hemisphere; LH: left hemisphere; IPS: information processing speed. * Frequency up to 2000 models.

## Data Availability

The datasets generated during and/or analyzed in the current study are available from the corresponding authors upon reasonable request.

## Supplementary Materials

The following are available online at https://www.mdpi.com/article/10.3390/jpm11111107/s1, MRI acquisition parameters, Table S1: Use of disease-modifying therapies at baseline, Table S2: Cognitive changes throughout the disease course, Table S3: Cognitive changes at the different phases of MS.
