# Supplementary material for: Dynamics and Predictors of Cognitive Impairment along the Disease Course in Multiple Sclerosis
Source: J Pers Med. 2021 Oct 28;11(11):1107. doi: 10.3390/jpm11111107 (PMC8624684; doi:10.3390/jpm11111107)
Supplement: Supplementary file 1 [file jpm-11-01107-s001.zip › jpm-1424355-supplementary.pdf]

Supplementary material

MRI acquisition parameters

We collected two different datasets (n1= 118 and n2 = 94) with the following acquisition parameters of the 3D-MPRAGE and 3D-FLAIR sequences, respectively. A 3D-structural image (n1) was acquired with TR = 1800 ms; TE = 3.01 ms; TI = 900 ms; 240 sagittal slices with 0.94 mm isotropic voxel size and a 256 × 256 matrix size; and a 3D-FLAIR with TR = 5000 ms; TE = 304 ms; TI = 1800 ms; 192 sagittal slices with 0.94 mm isotropic voxel size and a 256 × 256 matrix size. The other MRI dataset (n2) was obtained from other 3D-structural images with TR = 1970 ms; TE = 2.41 ms; TI = 1050 ms; 208 sagittal slices with 0.9 mm isotropic voxel size and a 256 x 256 matrix size; and a 3D-T2 FLAIR with TR = 5000 ms; TE = 393 ms; TI = 1800 ms; 208 sagittal slices with 0.9 mm isotropic voxel size and a 256 × 256 matrix size.

Table S1. Use of disease modifying therapies at baseline.

	**Patients using DMTs (n=111)**
Moderate-efficacy DMTs, n (%):	94 (85)
Teriflunomide	4 (4)
Interferon beta	68 (61)
Glatiramer acetate	19 (17)
Dimethyl fumarate	3 (3)
High-efficacy DMTs, n (%):	17 (15)
Fingolimod	7 (6)
Alemtuzumab	1 (1)
Natalizumab	9 (8)

DMTs: Disease Modifying Therapies.

Table S2. Cognitive changes throughout the disease course.

	n (n examinations)	β (95% CI)	p value
Global cognition z-score	212 (605)	-0.011 (-0.02 – 0.00)	0.058
Verbal memory z-score	212 (605)	-0.027 (-0.05 – -0.01)	0.007
Visual memory z-score	212 (605)	-0.018 (-0.03 – 0.00)	0.031
Attention-IPS z-score	212 (605)	0.008 (-0.01 – 0.02)	0.345
Semantic fluency z-score	210 (600)	-0.014 (-0.03 – 0.00)	0.043

Beta coefficients and 95% confidence intervals (CI) from age at MS onset, educational level, and sex adjusted mixed-effect models. IPS: information processing speed.

Table S3. Cognitive changes at the different phases of MS.

Cognitive domains	0-5 years			5-15 years			15-30 years		
	n (n examinations)	β (95% CI)	p value	n (n examinations)	β (95% CI)	p value	n (n examinations)	β (95% CI)	p value
Global cognition z-score	89 (223)	0.080 (0.04 – 0.12)	<0.001	121 (241)	-0.029 (-0.05 – - 0.01)	0.013	66 (141)	-0.031 (-0.06 – - 0.01)	0.021
Verbal memory z-score	89 (223)	0.083 (0.01 – 0.16)	0.037	121 (241)	-0.041 (-0.08 – 0.00)	0.047	66 (141)	-0.055 (-0.10 – - 0.01)	0.018
Visual memory z-score	89 (223)	0.061 (-0.01 – 0.13)	0.092	121 (241)	-0.041 (-0.08 – - 0.01)	0.024	66 (141)	-0.018 (-0.06 – 0.02)	0.359
Attention-IPS z-score	89 (223)	0.107 (0.05 – 0.16)	<0.001	121 (241)	0.003 (-0.03 – 0.03)	0.850	66 (141)	-0.035 (-0.07 – 0.00)	0.055
Semantic fluency z-score	88 (221)	0.035 (-0.03 – 0.09)	0.265	121 (240)	-0.020 (-0.05 – 0.01)	0.195	66 (139)	-0.028 (-0.06 – 0.01)	0.106

Beta coefficients and 95% confidence intervals (CI) from age at MS onset, educational level, level, and sex adjusted spline models with knots at 5 and 15 years of disease duration. IPS: information processing speed.
